# A New Record for Occurrence of *Symphodus bailloni* (Osteichthyes: Perciformes: Labridae) in the Western Black Sea Coast of Turkey

**DOI:** 10.1100/2012/615318

**Published:** 2012-04-19

**Authors:** Didem Göktürk, F. Saadet Karakulak, Nuran Ünsal, Abdullah E. Kahraman

**Affiliations:** ^1^Department of Fisheries Technology, Faculty of Fisheries, Istanbul University, Ordu Cad. No. 200, Laleli, 34470 Istanbul, Turkey; ^2^Department of Marine Biology, Faculty of Fisheries, Istanbul University, Ordu Cad. No. 200, Laleli, 34470 Istanbul, Turkey

## Abstract

The fish species *Symphodus bailloni* (Valenciennes, 1839) reported in the present study were collected between June 2010 and June 2011 from the western Black Sea coasts which were previously not recorded from the Black Sea coast of Turkey. A total of 717 specimens of *S. bailloni* were measured, ranging between 8.9 and 15.4 cm TL. Morphometrics, meristics, and diagnostic characteristics of the species are presented.

## 1. Introduction

*Symphodus *spp. are perciform fishes that belong to the Labridae family (wrasses) which is very large teleost family [1], third in number of species after Gobiidae and Serranidae [2]. Nelson [3] estimated the number of valid labrid species as 500 in about 60 genera; Allen [4] comprises 69 genera and *ca.* 500 species. Parenti and Randall [2] published an annotated checklist including 68 genera and 453 valid species. After the publication of the annotated checklist by Parenti and Randall [2], the species of Labridae increased from 453 to 504 and genera from 68 to 70 [5]. According to Hanel et al. [6] there are 580 species in 82 genera distributed in tropical and temperate marine waters around the world. Wrasses show strong sexual dichromatism [7, 8]. The color pattern may differ dramatically from juvenile to adult and with sex [2]. The species of the genus *Symphodus *have also generally an important sexual dimorphism [9]. In the past, the systematic of labrids was imprecise, with some genus misdescribed, and with the species' number changing constantly. This was due to the polymorphism that is common in this group of fishes [10]. Several studies mention the common misidentification of wrasses [11–14]. As suggested by Wheeler and Clark [11], *S. bailloni* can be misidentified/confused with *S. melops*. On the other hand, in previous studies, *S. melops* was recorded in the Aegean Sea [15–17].

The distribution of *Symphodus (Crenilabrus) bailloni* (Valenciennes, 1839) ranges, in the coastal waters of the eastern Atlantic, from Mauritania to the English Channel [18] and the southern North Sea, and throughout the western Mediterranean (off the coast of Spain and the Balearic Islands) [19]. Reuter [20] reported a specimen in the southern North Sea and Nijssen and Groot [21] had records of occurrence on the Dutch coast. Wheeler and Clark [11] described records of occurrence of the species around the British Isles, which are, briefly, near the Channel Islands in 1979 (1 specimen) and 1981 (2 specimens), on Swarte Bank in the southern North Sea in 1967 (1 specimen), off the Dutch coast (1 specimen each in 1967, 1968, 1972, and 1973), and off the coast of Galway, Ireland in 1982 (1 specimen). Although Wheeler and Clark [11] postulated that *Symphodus bailloni* may occur on the south and southwestern coasts of Britain, Dunn and Brown's report [22] appears to be the first actual record of the species on the south coast of England. *S. bailloni* is also reported to be a Lusitanian species in the Northern Europe fish fauna [11, 22]. Although there is no precise information available on its habitat [23], *S. bailloni* has been found at depths of 1–50 m mainly near rocks, in sea grass and eel-grass beds and maerl [11, 24, 25] and known as poorly studied species [22].

*Symphodus bailloni* (Valenciennes, 1839) reported in the present study were collected from the western Black Sea (coastal waters of Turkey) and it was not previously recorded from this area. The aim of this study is to report the new record for the occurrence of *S. bailloni* in the Black Sea.

## 2. Materials and Methods

**Figure 1:**
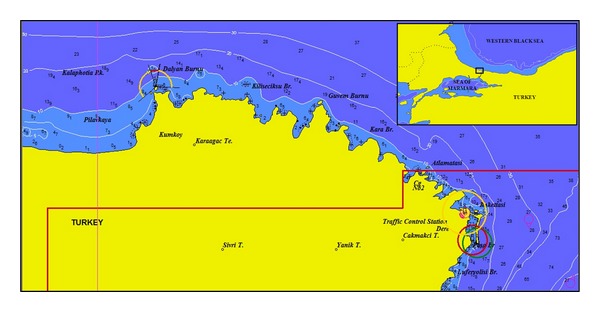
Study area: The Western Black Sea.

*S. bailloni* were captured during a gillnet selectivity survey in the Western Black Sea. Study was carried out monthly from June 2010 to June 2011 in the Western Black Sea (Figure 1). The sampling was conducted by gillnets (100 m long—17, 18, 20 mm nominal bar length) on rocky, sandy, and muddy bottoms within 4.5 and 28 m depths. Surface water temperature, salinity, and dissolved oxygen were recorded once during each tow. The specimen of *S. bailloni *was identified following the nomenclature reported in Whitehead et al. [26] as well as the morphometric measurements and meristic counts of fish specimens. Total length (TL) was measured to the nearest full cm below and the measurements of head length and body depth taken with a digital calliper to the nearest 0.1 mm. Lateral line scales and gill rakers were counted using an image analysis system (Leica DFC295 camera attached Leica S8APO stereomicroscope with LAS software). The physical and chemical characteristics in the studied area were monitored including, dissolved oxygen, salinity and temperature. Salinity values ranged from 12.3 ppt to 15.4 ppt. Dissolved oxygen values ranged from 6.44 to 11.84 mg/L. Temperature values varied between 7.2°C and 29.1°C.

## 3. Results

**Figure 2:**
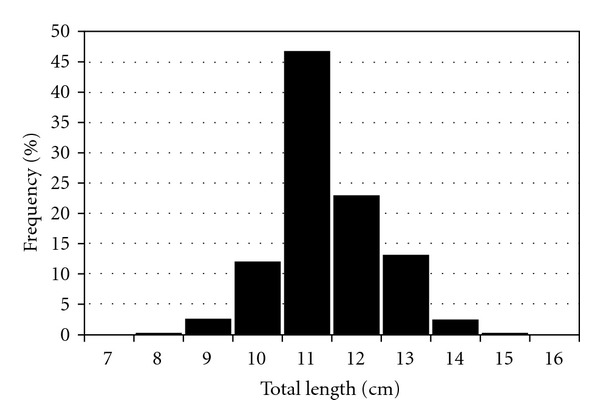
Total length-frequency distribution of *Symphodus bailloni*.

**Figure 3:**
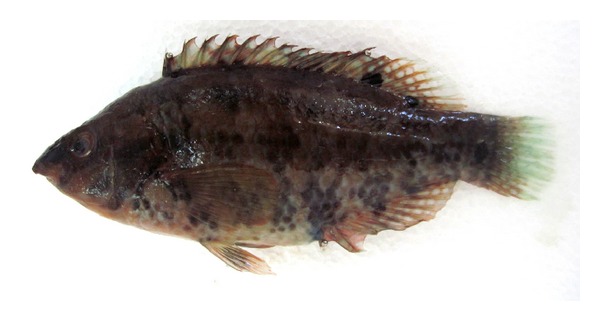
*Symphodus bailloni*, 13.1 cm standard length, from western Black Sea coast of Turkey.

A total of 717 specimens of *S. bailloni* were measured, ranging between 8.9 and 15.4 cm TL (Figure 2). 20 specimens were chosen to make the morphometric measurements and meristic counts of *S. bailloni* and done based on Whitehead et al. [26]. The individuals had all the distinguishing features of the species, including metric and meristic characters that agree with those reported for the species. For field identification, we found that three anal spines, a serrated preoperculum, a dark blotch at the base of the beginning of the soft dorsal fin, and a smaller dark spot at the end of the soft dorsal fin, a dark spot at the caudal peduncle are characteristic features for *S. bailloni*. Distance between base of second dorsal spiny ray and lateral line is not smaller than half length of soft part of dorsal fin. This is the main difference point of *S. bailloni* from *S. roissali*. As seen in Figure 3 general appearances and some diagnostic features of *S. bailloni* are as follows.

**Figure 4:**
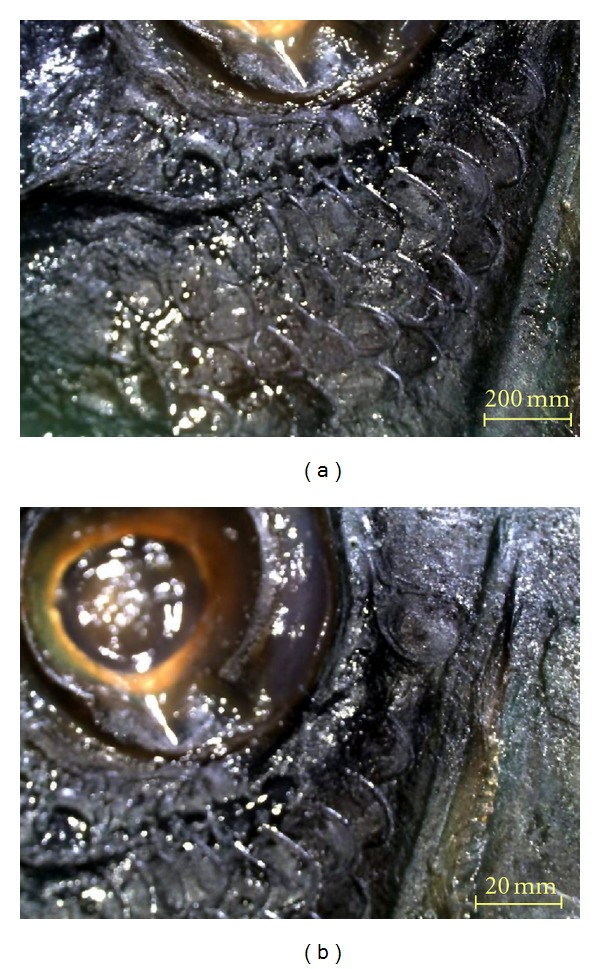
(a) Rows of scales on cheek. (b) Rows of scales behind eye.

**Figure 5:**
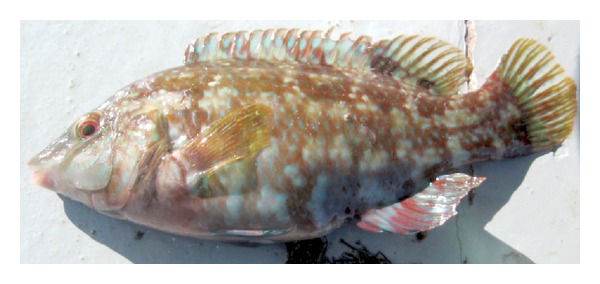
*Symphodus bailloni*, from western Black Sea coast of Turkey (on board).

Symphodus bailloni (Valenciennes, 1839).
Materials examined: June 2010–June 2011, 717 specimens, Length: 8.9–15.4 cm TL.
Diagnostic characteristics (20 specimens were chosen): head length is shorter than body depth. Preorbital shorter than postorbital. Teeth rather small (3–5/5–8). Dorsal fin rays XV + 9-10; anal fin rays III 9-10; pelvic fin rays 13-14; ventral fin rays I + 5; caudal fin rays 15.
Scales along lateral line 33–37, rows of scales on cheek 2-3 (Figure 4(a)), behind eye 1 (Figure 4(b)). 12–14 gillrakers on the first branchial arch. 67–86 cephalic pores on snout. Vertebrae 31–33.
Color: a dark spot on caudal peduncle and another brown-black spot on beginning of soft part of dorsal fin, and a smaller dark spot at the end of the soft dorsal fin. 5 vertical dark brown patches on upper part of flanks, reaching belly and anal fin. Usually, the color of body reddish brown or greenish-reddish brown. In our case generally the variation of color depends on the environment especially in laboratory or on board. The photo of *S. bailloni* which is shown in Figure 5 was taken from the surve on board.

## 4. Discussion

Our aim is to report the *Symphodus bailloni*, which is unrecorded fish species for Western Black Sea. Of all the inland seas such as the White Sea, the Baltic Sea, and the Mediterranean Sea, the Black Sea is mostly isolated from the world oceans. It is a semiclosed basin with relatively great depths, and high bioproductivity of the shelf zone [27–29]. The most remarkable feature of the Black Sea is that nearly 87 percent of the Black Sea water volume is anoxic [28, 30, 31] and it contains high levels of the hydrogen sulphide [32–34]. Compared with the Sea of Marmara, the Aegean Sea, and the Mediterranean Sea. It has lower salinity levels [31, 32], salinity ranges from 17 to 22%; in the Black Sea [35]. Fresh water inputs coming from the big rivers and exchange of the Mediterranean water via the Bosphorus are critical elements in the hydrography and ecosystem of the Black Sea [30].

The Black sea is inhabited by 168 species, from which 144 are typically marine ones, and 24 diadromous or partly anadromous [28, 36, 37]. In the Turkish coasts of the Black Sea, Erazi [38] reported 128 fish species, Kocataş et al. [39] 150 species, Mater and Meriç [16] 138 species, Öztürk [40] 140 species, and Bilecenoğlu et al. [41] 151 species. The composition of the Black Sea ichthyofauna has changed in response to alterations in living conditions in the sea. Some of the changes had an impact on coastal and shelf waters, others on the pelagic zone, affecting common and rare species, fry and adults, commercial and noncommercial species [30]. For several decades, the ecosystem of the Black Sea has been changing mainly from the human activities [42–48], and consequently the new species enter to this basin [49–52]. For the time being, there are about 60 alien species in the Black Sea [53].

The habitat preferences of *S. bailloni* are poorly known. It was reported that *S. bailloni* was caught as the most common species in the particular habitat of the Solent Estuaries (English Channel) where the salinity levels were low [22]. In addition, Rodrigues [23] indicated that the types of habitat at Professor Luiz Saldanha Marine Park (Portugal) were rocky, sandy, and sea-grass beds. In this study, the habitat where our research was carried out have low salinity levels as well as several bottom types such as rocky, muddy, sandy, and partly sea-grass beds. Similarly, Dunn and Brown [22], and Rodrigues [23] indicated in their studies that the types of habitat were similar to those of our habitat. Therefore, it can be safely concluded that this species prefer the habitat in which the salinity levels are low.

There are no any comprehensive studies regarding the ichthyofauna, especially on labridae, of the Turkish coasts of the Black Sea. This study is the first record for *S. bailloni* in the Black Sea, and it was observed that *S. bailloni* is the most abundant species, of all the other *Symphodus* species (*S. ocellatus, S. roissali, *and* S. tinca*). It has been well known that many exotic species such as* Mnemiopsis leidyi*, *Mugil soiuy*, *Rapana thomasiana*, and *Balanus improvisus, *has entered and distributed/spread in the Black Sea due to human activities (ballast waters, aquaculture, etc.). In our study, it was found that the occurrence of relatively high numbers of *S. bailloni* in the Western Black Sea is noteworthy. Thus, this species has been considered to be placed in this region as a resident population. This report appears to be the first record of the species on the north coast of Turkey (Black Sea). Furthermore, there is a lack of information on age and growth, sexual maturity, diet composition, and reproduction biology of this species. It is clear that the biological and ecological studies on *S. bailloni* to be carried out in the future will contribute to the Black Sea biodiversity.

## References

[1] Darwall WRT, Costello MJ, Donnelly R, Lysaght S (1992). Implications of life-history strategies for a new wrasse fishery. *Journal of Fish Biology*.

[2] Parenti P, Randall JE (2000). An annotated checklist of the species of the Labroid fish families Labridae and Scaridae. *Ichthyological Bulletin of the JLB Smith Institute of Ichthyology*.

[3] Nelson JS (1994). *Fishes of the World*.

[4] Allen G (1999). *Marine Fishes of Tropical Australia and South-East Asia: A Field Guide for Anglers and Divers*.

[5] Parenti P, Randall JE (2011). Checklist of the species of the families Labridae and Scaridae: an update. *Smithiana Bulletin*.

[6] Hanel R, Westneat MW, Sturmbauer C (2002). Phylogenetic relationships, evolution of broodcare behavior, and geographic speciation in the wrasse tribe Labrini. *Journal of Molecular Evolution*.

[7] Carpenter KE (2002). The living marine resources of the Western Central Atlantic. *Bony Fishes Part 2 (Opistognathidae to Molidae), Sea Turtles and Marine Mammals*.

[8] Carpenter KE, Niem VH (2001). The living marine resources of the Western Central Pacific. *Bony Fishes Part 4 (Labridae to Latimeriidae), Estuarine Crocodiles, Sea Turtles, Sea Snakes and Marine Mammals*.

[9] Voss J (1775). A propos de quelques poissons de la Méditerranée: le genre Symphodus Rafinesque, 1810: Symphodus (Crenilabrus) melops L., Symphodus ocellatus Forskål. *Revue fr. Aquariol*.

[10] Lejeune P (1802). Étude écoéthologique des comportements reproducteurs et sociaux des Labridés méditerranéens des genres Symphodus Rafinesque, 1810, et Coris Lacepede. *Cahiers d’Ethologie Appliquee*.

[11] Wheeler A, Clark P (1984). New records for the occurrence of Crenilabrus bailloni (Osteichthyes: Perciformes: Labridae) in the waters of northern Europe. *Journal of the Marine Biological Association of the United Kingdom*.

[12] Couch J (1868). *A History of the Fishes of the British Isles*.

[13] Yarrell W (1836). *A History of British Fishes*.

[14] Day F (1880–1884). *The Fishes of Great Britain and Ireland*.

[15] Fischer W, Bauchot ML, Schneider M (1987). Fiches FAO d’identification des espèces pour les besoins de la pêche: Vertèbrès. *(Rèvision 1) Mèditerranèe et Mer Noire. Zone de Pêche 37*.

[16] Mater S, Meriç N, Kence A, Bilgin CC (1996). Deniz balıkları (Marine fishes). *The Species List of Vertebrates in Turkey*.

[17] Mater S, Bilecenoğlu M, Demirsoy A (1999). Türkiye deniz balıkları (Marine Fishes of Turkey). *Genel Zoocoğrafya ve Türkiye Zoocoğrafyası*.

[18] Quignard JP (1966). Recherches sur les Labridae (Poissons Téléostens Perciforms) des côtes européennes-Systématique et Biologie. *Naturalia Monspeliensia. Série Zoologique*.

[19] Sayer MDJ, Treasurer JW, Sayer MDJ, Treasurer JW, Costello MJ (1996). North European wrasse: identification, distribution and habitat. *Wrasse: Biology and Use in Aquaculture*.

[20] Reuter JH (1967). A first report of Bailloni’s wrasse in the North sea. *Netherlands Journal of Sea Research*.

[21] Nijssen H, Groot SJ (1974). Catalogue of fish species of the Netherlands. *Beaufortia*.

[22] Dunn MR, Brown MJ (2003). The occurrence of *Symphodus bailloni* on the south coast of England. *Journal of the Marine Biological Association of the United Kingdom*.

[23] Rodrigues DD (2010). *Habitat associations and behaviour of wrasses of the genus Symphodus (Rafinesque, 1810) at the Arrábida Marine Park, Portugal*.

[24] Bauchot ML, Quignard JP, Hureau JC, Monod T (1973). Labridae. *Checklist of the Fishes of the North-Eastern Atlantic and of the Mediterranean*.

[25] Quignard JP, Pras A, Whitehead PJP, Bauchot ML, Hureau JC, Nielsen J, Tortonese E (1986). Labridae. *Fishes of the Northeastern Atlantic and the Mediterranean*.

[26] Whitehead PJP, Bauchot ML, Hureau JC, Nielsen J, Tortonese E (1986). *Fishes of the North-eastern Atlantic and the Mediterranean*.

[27] Rass TS (1992). Changes in the fish resources of the Black Sea. *Oceanology*.

[28] Prodanov K, Mikhailov K, Daskalov G (1997). *Environmental Management of Fish Resources in the Black Sea and Their Rational Exploitation*.

[29] Shiganova TA, Bulgakova YV (2000). Effects of gelatinous plankton on Black Sea and Sea of Azov fish and their food resources. *ICES Journal of Marine Science*.

[30] Zaitsev YU, Mamaev VO (1997). *Biological Diversity in the Black Sea: A Study of Change and Decline*.

[31] Murray JW, Stewart K, Kassakian S, Krynytzky M, DiJulio D, Yanko-Hombach V, Gilbert AS, Panin N, Dolukhanov PM (2007). Oxic, suboxic and anoxic conditions in the Black Sea. *The Black Sea Flood Question: Changes in Coastline, Climate and Human Settlement*.

[32] Murray JW (1991). The 1988 Black Sea oceanographic expedition: introduction and summary. *Deep Sea Research*.

[33] Oguz T, Ducklow HW, Malanotte-Rizzoli P (2000). Modeling distinct vertical biogeochemical structure of the black sea: dynamical coupling of the oxic, suboxic, and anoxic layers. *Global Biogeochemical Cycles*.

[34] Oguz T (2002). The role of physical processes controlling oxycline and suboxic layer structures in the Black Sea. *Global Biogeochemical Cycles*.

[35] Oguz T, Ivanov LI, Besiktepe S, Ivanov LI, Oguz T (1998). Circulation and hydrographic characteristics of the Black Sea during July 1992. *Ecosystem Modeling as a Management Tool for the Black Sea*.

[36] Prodanov K, Ivanov L, Dencheva K (1993). *Biodiversity of the Ichthyofauna in the Bulgarian Black Sea Waters*.

[37] Rass TS (1987). Present notions about taxonomic composition and changes in ichthiophauna of the Black Sea. *Intel® vPro*™* Technology*.

[38] Erazi RAR (1942). Marine fishes found in the sea of marmara and in the bosphorus. *Revue de la Faculté des Sciences de l’Université d’Istanbul*.

[39] Kocataş A, Ergen Z, Mater S, Kence A (1987). Marine fauna. *(Programme Coordinator) Biological Diversity in Turkey*.

[40] Öztürk B (1999). *Black Sea Biological Diversity Turkey*.

[41] Bilecenoğlu M, Taskavak E, Mater S, Kaya M (2002). *Checklist of the Marine Fishes of Turkey (Zootaxa 113)*.

[42] Bologa AS (1986). Planktonic primary productivity of the Black Sea: review. *Thalassia Jugosl*.

[43] Chirea R, Gomoiu MT (1986). Some preliminary data on the nutrient influx into the western Black Sea. *Cercetari marine-Marine Research, IRCM Constanta*.

[44] Mee LD (1992). The Black Sea in crisis: a need for concerted international action. *Ambio*.

[45] Mee LD, Topping G (1999). *Black Sea Pollution Assessment*.

[46] Zaitsev YU, Öztürk B (2001). *Exotic Species in the Aegean, Marmara, Black, Azov and Caspian Seas*.

[47] Wonham MJ, Carlton JT, Ruiz GM, Smith LD (2000). Fish and ships: relating dispersal frequency to success in biological invasions. *Marine Biology*.

[48] Gokturk D (2005). *Ballast water samplings in Istanbul ports*.

[49] Tolmazin D (1985). Changing coastal oceanography of the Black Sea. I: Northwestern shelf. *Progress in Oceanography*.

[50] Ivanov L, Beverton RJH (1985). *The Fisheries Resources of the Mediterranean-Part two: Black Sea. Studies and Reviews*.

[51] Caddy JF, Griffiths RC (1990). A perspective on recent fishery-related events in the Black Sea. *Studies and Reviews*.

[52] Zaitsev YUP (1993). Fisheries and environment studies in the Black Sea system. Part 2: impact of eutrophication on the Black Sea fauna. *Studies and Reviews*.

[53] Arvanitidis C, Eleftheriou A, Berghe EV Electronic conference on Marine Biodiversity in the Mediterranean and the Black Sea-summary of discussions.

